# Self-compassion is associated with less stress and depression and greater attention and brain response to affective stimuli in women managers

**DOI:** 10.1186/s12905-018-0685-y

**Published:** 2018-11-27

**Authors:** Fernanda B. C. Pires, Shirley S. Lacerda, Joana B. Balardin, Bruna Portes, Patrícia R. Tobo, Carla R. C. Barrichello, Edson Amaro, Elisa H. Kozasa

**Affiliations:** 1 Hospital Israelita Albert Einstein, Av. Albert Einstein, 627/701, São Paulo, SP Brazil; 2 Natura Cosméticos S.A, Rodovia Anhanguera km 30.5, Cajamar, SP, Brazil

**Keywords:** Self-compassion, fMRI, Precuneus, Emotional reactivity, Empathy, Women

## Abstract

**Background:**

Women have been assuming more responsibilities and higher positions in major companies, which exposes them to high levels of stress. Higher perceived work stress is related to higher emotional reactivity. Difficulties with emotional regulation can lead to anxiety and mood disorders, which are more prevalent in women than men. Indeed, women leaders are more likely to experience emotional fatigue than men due to excessive empathy. Our aim was to evaluate the associations between self-compassion (SC) scores to depression symptoms, perceived stress and mindfulness, as well as with brain responses to high-arousal unpleasant and pleasant pictures from the International Affective Picture System (IAPS), as measured through functional Magnetic Resonance Imaging (fMRI) in women managers.

**Methods:**

Forty-six participants were selected for the study. All participants filled the Self Compassion Scale (SCS), Beck Depression Inventory (BDI), Mindful Awareness Attention Scale (MAAS) and the Perceived Stress Scale (PSS). After that they were scanned during an fMRI affective response paradigm. Correlation analysis were performed among these variables.

**Results:**

Our data suggest that women with higher SC scores respond to affective stimuli with higher activation of the precuneus (a brain region related to self-referential processing), lower levels of stress and depression and show greater attention in everyday activities.

**Conclusion:**

SC may be an important characteristic for women leaders because of its association with higher sensitivity to emotional stimuli and mindfulness. These skills may allow them to be more aware of others while being less susceptible for stress and depression symptoms.

**Electronic supplementary material:**

The online version of this article (10.1186/s12905-018-0685-y) contains supplementary material, which is available to authorized users.

## Background

Women have been increasingly assuming more responsibilities and higher positions in major companies, which exposes them to relatively higher levels of stress [1]. Higher perceived work stress is related to higher emotional reactivity, higher perseveration and lower interest at work. Work stress and resilience depend on individual characteristics that may lead to better stress management [2].

Difficulties with emotional regulation can lead to anxiety and mood disorders, which are more prevalent in women than men. An event-related potential (ERP) study examining sex differences in emotional reactivity during passive viewing of unpleasant images showed that women presented early emotional reactivity and had greater emotional appraisal towards unpleasant emotional stimuli [3].

Populations particularly prone to excessive stress, emotional reactivity and empathy fatigue include health care professionals [4], social and aid workers, and leaders [5].

Some evidence suggests that women tend to be more aware of others’ emotions and needs than men, who are usually more aware of their own feelings [6]. Indeed, women leaders are more likely to experience emotional stress or fatigue than men due to excessive empathy [7]. Also, professionals who have more difficulty regulating their unpleasant arousal and identifying their emotions tend to be more frustrated and emotionally exhausted, while those who have more awareness and are better able to regulate their own emotions can express compassion and obtain more satisfaction in helping others without distress [7, 8]. This suggests that to regulate and identify one’s own emotions one must first be aware and mindful of one’s own needs and limits, which is at the core of developing self-compassion (SC) [9].

Compassion is defined as having deep perception of the suffering of others accompanied by the wish to immediately relieve it and offer care, as well as understanding without judgment or a sense of pity. SC is compassion directed towards oneself in situations of difficulty or suffering. Unlike self-esteem, SC does not depend on external conditions and is associated with greater resilience and the ability to relate more gently with oneself [10]. Finally, SC allows one to view one’s own faults as part of the universal human experience and to see those experiences as being part of the larger human experience instead of with feelings of isolation or disconnection [11]. This more positive view of the world can protect the individual from negative and depressive thinking [12].

There is an increased research interest in the relation among SC, mindfulness and affect. In a large group of adults representative of the Dutch population in age and gender distributions (*N* = 1736), a study examined the predictive value of mindfulness and SC on depressive symptoms and affect. The authors studied the Five Facets of Mindfulness Questionnaire (FFMQ) (observe, describe, act with awareness, non-judgment, and non-reactivity) and two facets of the Self-Compassion Scale (SCS) and found that three of the five FFMQ facets and SCS negative items significantly predicted both depressive symptoms and negative affect, with SCS negative items (having a harsh attitude towards oneself) and ‘act with awareness’ as the strongest predictors [13].

In another study, Arch et al. [14] evaluated whether a brief training in SC would moderate biopsychological responses to social stress in women (relative to attention (placebo) and no-training control conditions). Relative to the control groups, the women who received the training showed reduced sympathetic (salivary alpha-amylase), cardiac parasympathetic, and subjective anxiety responses (measured with the Trier Social Stress Test; TSST), as well as increased SC under threat. Therefore, SC seems to be critical in diminishing social stress and negative psychological and biological effects [14].

Neuroimaging studies have implicated regions of the default mode network (DMN) in the processing of social emotions [15]. The DMN is generally active when the brain is in a state of wakeful rest and the individual is not focused on the outside world or engaged in any particular task. DMN regions are activated in tasks that require interaction with other people, perceiving and interpreting others’ emotions, empathizing, understanding, and judging others’ intentions [16]. One of the key structures in the DMN is the precuneus.

Whole-brain cross-modal analyses showed that activation in the precuneus distinguished between pleasant versus unpleasant conditions [17].

To date, little is known regarding the brain regions involved in SC, as well as its relation to other cognitive and psychological characteristics and its variation across individuals. Therefore, in this study we aimed to correlate women’s SC scores to depression symptoms, perceived stress and mindfulness, as well as with brain responses to high-arousal unpleasant and pleasant pictures from the International Affective Picture System (IAPS) [18], as measured through functional Magnetic Resonance Imaging (fMRI).

We hypothesized that women with higher SC scores would present lower stress and depression scores, as well as increased activation in brain regions associated with affective responses and self-other awareness in the unpleasant>neutral and unpleasant>pleasant contrast.

## Methods

### Participants

The participants in this study were women sales and administrative managers in a Brazilian multinational cosmetics company who had at least 15 years of education and stress complaints. Of 167 women who were invited, 99 volunteered to participate in the study. Of those, 23 were not able to attend the scheduled meetings and the remaining 76 were evaluated and signed the informed consent. Of the 76, 21 did not meet criteria for undergoing fMRI (2 had metal orthodontic pieces, 9 were left-handed, 6 were claustrophobic, 2 had neurological problems and 2 were pregnant). Four of the women who did not meet fMRI criteria also had psychiatric symptoms and were recommended for treatment. Of the 55 women who underwent fMRI, nine were excluded for problems with image acquisition or behavioral testing, leaving a total of 46 study participants (mean age 43.26, SD 8.36). All participants were screened by an experienced clinical physician and none met criteria for starting psychiatric treatment. Four women who had used antidepressants at a stable dose for more than three months were included in the study.

The study was approved by the ethics committee at Hospital Israelita Albert Einstein (Protocol number: 38662314.8.0000.0071).

All participants filled the SCS, Beck Depression Inventory (BDI), Mindful Awareness Attention Scale (MAAS) and the Perceived Stress Scale (PSS). After that they were scanned during an fMRI affective response paradigm.

### Instruments

#### **The international affective picture system (IAPS)** [18]

This internationally accessible database contains standardized color photographs of emotionally-evocative scenes from thousands of diverse categories. In this study, all participants viewed the same 45 pictures (15 unpleasant, 15 neutral and 15 pleasant). We chose the themes that elicited the highest unpleasant and pleasant arousal levels, respectively.

### **Self-compassion scale (SCS)** [19, 20]

This scale contains 26 items that measure respondents’ attitudes toward themselves with respect to personal flaws, failures, and painful events. It includes five items related to self-kindness, five reverse-scored items related to self-judgment, four items related to perceptions of common humanity, four reverse-scored items related to perceived isolation, four items related to mindfulness, and four reverse-scored items related to over-identification with emotions. Responses are given on a 5-point Likert-type scale (1 = *Almost never*; 5 = *Almost always*). Internal consistency (Cronbach’s alpha) of 26 items of SCS-Brazil was 0.92 [20].

### **Perceived stress scale (PSS)** [21, 22]

This scale contains 10 sensation-related items that measure the degree to which individuals perceive situations as stressful and how unpredictable, uncontrollable, and overloaded they consider their lives to be. The PSS is a general scale that may be used with different age groups because it does not contain context-specific questions. The internal consistency of the Brazilian version of PSS was 0.83 [22].

### **Beck depression inventory (BDI)** [23, 24]

This inventory consists of 21 descriptive statements of depression symptoms and respondents answer on a scale of 0–3. The internal consistency measured by the Cronbach’s alpha for the Brazilian version of BDI was 0.88 [24].

### **Mindful attention awareness scale (MAAS)** [25, 26]

With this 15-question scale, respondents indicate how frequently they experience certain attention and awareness levels in everyday situations, on a scale of 1–6. The Cronbach’s alpha coefficient for the Brazilian version of MAAS was 0.83 [26].

### fMRI paradigm

Prior to fMRI, participants were familiarized with pictures from the IAPS. During the scan, each picture was presented for 2 s and participants were asked to rate their valence by pressing a button (for a maximum of 2 s). Picture presentation was cued by a yellow square in the middle of the screen presented for 500 ms. All participants viewed 15 pictures from each of the three conditions (neutral, pleasant, and unpleasant, according to validated IAPS ratings). A randomly jittered inter-trial interval (7–13 s) was used to sample the hemodynamic response at different time points.

### Image acquisition

Image acquisition (3.0 T MR system—Siemens Tim Trio, 12ch head coil), visual stimuli presentation, and subject response were synchronized (NNL systems, www. nordicneurolab.com). The fMRI acquisition was based on whole-brain T2*-weighted echo planar images (EPI). The acquisition parameters were EPI GRE T2–BOLD PACE: TR = 2000 ms, TE = 50 ms, 32 slices, 3.3 mm of slice thickness, 0.5 mm of interslice gap, FOV = 200 mm and matrix 64 Å~ 64, 3 mm3 voxels, with 354 volumes (duration: 11m48s). For optimization of the event-related design, we used a genetic algorithm [27] to estimate randomly jittered inter-trial intervals (7–13 s). The pleasant and unpleasant pictures with the highest arousal scores were chosen for this study.

### Image processing

The fMRI data processing was carried out using fMRI Expert Analysis Tool (FEAT) Version 5.0.8, part of FSL (www.fmrib.ox.ac.uk/fsl/) [28]. Functional data were registered to the high resolution structural image and the structural image was registered to the MNI152 standard space using FLIRT [29, 30]. The following pre-statistics processing was applied: motion correction MCFLIRT [30]; non-brain removal using BET [31]; spatial smoothing (FWHM = 5 mm); grand-mean intensity normalization of the entire 4D dataset by a single multiplicative factor; high-pass temporal filtering (Gaussian-weighted least-squares straight line fitting, with sigma = 50.0 s). The activation maps were produced using the general linear model (GLM) using FILM routines, which is based on semi-parametric estimation of residual autocorrelation [32]. At the single-subject level, a regressor of interest for each trial type was created (i.e., pleasant, neutral, and unpleasant valences modelling the 2 s visualization period). All regressors were convolved with a gamma hemodynamic response function. Individual contrasts were computed for unpleasant vs. neutral and unpleasant vs. pleasant trials. At the second-level analysis, we used a whole-brain multiregression analysis to investigate the relation between self-compassion traits and BOLD signal change for the critical contrasts. We also controlled for the potential confounding effect of severity of perceived stress (covariate), since all participants had stress complaints. All the statistical images were thresholded by using Gaussian random field-based cluster inference with a threshold of Z > 2.3 at the voxel level and a corrected cluster significance threshold of *p* < 0.05.

### Other variable statistics

Descriptive statistics and Pearson correlation analyses were conducted among SCS, MAAS, BDI and PSS scores. Additionally, a stepwise multiple regression analysis with precuneus activation as a dependent variable and the MAAS, SCS, BDI and PSS scores was performed.

## Results

### Sample characteristics

The mean, standard deviation, median, minimum and maximum values of age, SCS, MAAS, PSS and BDI scores are presented in Table 1.

**Table 1:** Sample characteristics for the variables measured

	Mean	SD	Median	Minimum	Maximum
Age (years)	43.26	8.36	42.00	30	63
SCS (scores)	87.43	15.51	85.50	52	120
MAAS (scores)	51.67	12.94	54.00	22	86
PPS (scores)	20.43	6.82	19.00	9	34
BDI (scores)	13.48	9.32	11.00	1	41

*SCS* Self-Compassion Scale, *MAAS* Mindful Attention Awareness Scale, *PPS* Perceived Stress Scale, *BDI* Beck Depression Inventory

A stepwise multiple regression model with precuneus activation as a dependent variable and the MAAS, SCS, BDI and PSS scores as independent variables showed that the SCS score was the only significant predictor for precuneus activation (β = 0.008, *p* < 0.001).

### 1.2 Effects of self-compassion scores on emotional reactivity-related activation

**Fig. 1:**
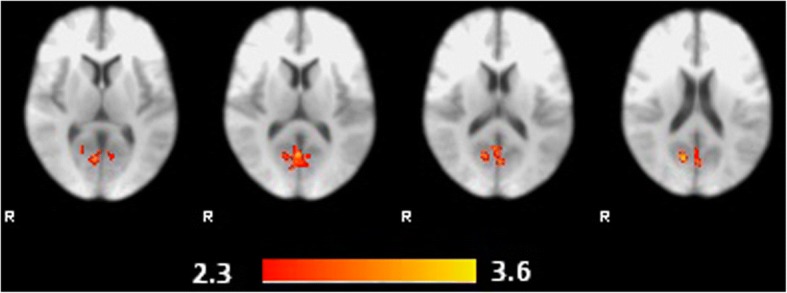
Brain regions activated for the unpleasant>pleasant contrast correlated with the self-compassion scores (cluster threshold Z > 2.3, *p* < 0.05 corrected)

**Fig. 2:**
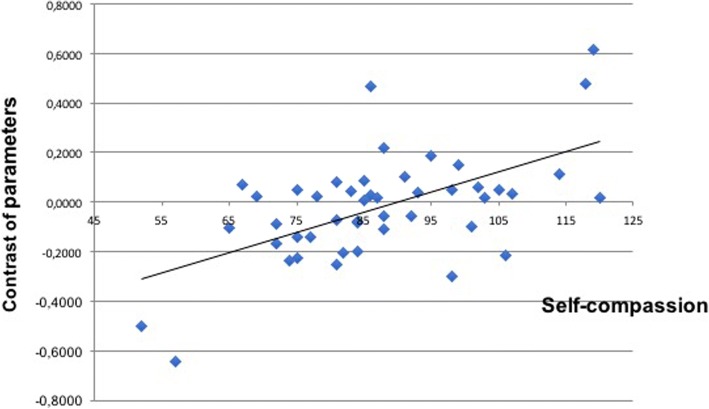
Correlation between self-compassion scale and precuneus activation in the unpleasant>pleasant contrast

Self-compassion scores were significantly correlated with signal changes in the unpleasant vs. pleasant contrast in a cluster encompassing the precuneus cortex. The MNI coordinates are presented in Table 2; Fig. 1 shows the cluster encompassing the precuneus cortex and Fig. 2 presents the correlation graph between the variables. There were no significant findings for the unpleasant vs. neutral contrast. The group average activation maps for the main contrasts of interest in the current study were presented in the Additional file 1.

**Table 2:** Region activated in the unpleasant>pleasant contrast, which was also correlated with self-compassion

Region	peak Z-value	MNI Coordinates				
		x	y	z	Cluster size	Cluster *p*-value
Precuneus	3.7	12	−66	18	390	< 0.001

There were also significant correlations between MAAS, SCS, BDI and PSS scales (Table 3).

**Table 3:** Correlation between self-compassion, mindfulness, stress and depression symptoms

		MAAS	PSS	BDI
SCS	r	0.557**	−0.625**	−0.581**
MAAS	r		−0.667**	−0.521**
PSS	r			0.719**

**p < 0.001, *SCS-* Self-Compassion Scale, *MAAS*- Mindful Awareness Attention Scale, *PSS*- Perceived Stress Scale, *BDI*- Beck Depression Inventory

## Discussion

For the participants studied in the current work, SC was positively correlated with mindfulness and with activation in the precuneus (a DMN region) in the high-arousal unpleasant>high arousal pleasant picture contrast, and negatively correlated with depression and perceived stress. Therefore, compared with women with low SC scores, women with higher SC scores presented increased activation in the precuneus for the high-arousal unpleasant>high arousal pleasant contrast, as well as higher mindfulness and lower perceived stress and depression.

Previous work has shown an association between the DMN and regions involved in social, affective and introspective processes [33]. Overlapping regions are considered the social-affective part of the DMN. The authors mapped the underlying brain network formed by these regions and regions strongly connected to them and observed that the posterior cingulate/precuneus and dorsomedial prefrontal cortex were associated with mentalizing, self-reference and autobiographic information.

In Otti et al. [34], healthy participants underwent a resting-state fMRI and the presentation of pictures of human limbs in painful and non-painful situations. After the exam, participants scored the visual stimuli in terms of pain intensity from the first-person perspective. Painful figures led to a relative increase in activity of DMN regions compared to no painful, suggesting that identifying with another’s pain influences the response of the DMN. The DMN has a self-referential mode and is related to our response to the environment.

In an fMRI experiment, Baucom et al. [35] evaluated brain activity during the presentation of pictures with high or low arousal levels and neutral, positive or negative valence. Voxel clusters involved in valence and arousal interpretation included the inferior temporal gyrus, lentiform nucleus, medial prefrontal cortex, middle occipital gyrus, middle temporal gyrus, parahippocampus, postcentral gyrus, and precuneus.

Using multivariate pattern analyses (MVPA), Saarimaki et al. [36] classified brain activity patterns for six basic emotions (disgust, fear, happiness, sadness, anger, and surprise) in an fMRI task where participants viewed movies or engaged in mental imagery. Activity patterns generalized between conditions and across individuals. The most active brain regions included medial and inferior lateral prefrontal cortices, frontal pole, precentral and postcentral gyri, precuneus, and posterior cingulate cortex [36].

The precuneus has been associated with the processing of affective valence [17] and social emotions [37]. In our study, activity in the precuneus may reflect the detection of the difference in valence between high arousal unpleasant and pleasant pictures and the recognition of emotions induced by those pictures (especially the unpleasant pictures involving human suffering).

While no previous studies have investigated the neurobiology of SC, there are several studies about other types of compassion. Engen and Singer [38] proposed compassion-meditation as a strategy to attenuate or modulate emotional stimuli. However, this attenuation could be problematic in the case of emotions elicited by the suffering of others, as it could lead to reduced emotional connectedness. In their fMRI experiment, Engen and Singer presented videos showing people in distress to 15 expert practitioners of compassion-meditation who either viewed the videos passively or used compassion-meditation or reappraisal to modulate their emotional reactions. Compassion increased positive affect and reappraisal decreased negative affect. Relative to the other strategies, compassion increased activation in regions involved in affiliation, positive affect and reward processing, including ventral striatum and medial orbitofrontal cortex.

According to Weng et al. [39], the cultivation of compassion involves the development of altruism, which increases the recruitment of brain systems related to executive and emotional regulation, the comprehension of suffering of others and reward (dorsolateral prefrontal cortex (DLPFC), inferior parietal cortex, DLPFC connectivity with the nucleus accumbens). The fact that we did not observe compassion-related regions in our study suggests that SC, with its self-referential nature, is a different construct than general compassion.

In a study by Krieger et al. [40], depressed (*N* = 142) and non-depressed (*N* = 120) individuals from a community sample completed self-report measures. Relative to never-depressed individuals, the depressed patients reported lower levels of SC. Furthermore, SC was negatively related to depressive symptoms, rumination and cognitive and behavioural avoidance in depressed outpatients. Thus, rumination and cognitive and behavioural avoidance mediated the relationship between SC and depression [40]. In line with these results, our own study showed a negative correlation between depression and SC.

In a structural equation modelling study, 36 and 67% of the variance in well-being were explained by self-compassion and attitudes toward aging, according to Brown et al. [41]. In this sample of 517 midlife women, SC was an important predictor of attitudes toward physical change, psychosocial loss and growth. Midlife women were a group strongly represented in our own study.

Also, in a group of women, brief SC training diminished salivary alpha-amylase and subjective anxiety responses and increased self-compassion under threat, relative to the control groups. Therefore, training SC is a promising method for reducing social stress and negative psychological and biological effects in women [14].

The psychological scale results also showed that women with higher SC presented less perceived stress and higher mindfulness, indicating that SC may promote wellbeing [42, 43]. Overall, our data suggest that SC may allow individuals to have emotional sensitivity to unpleasant situations without losing mindfulness or becoming distressed.

### Limitations

Our study focused on women in leading positions, as this group is more prone than men to experience exhaustion, stress, depression, and fatigue [7, 44]. Future research should determine the generality of these findings to a wider range of populations and contexts. Also, as this was a cross-sectional study, it does not allow us to understand factors that may improve SC.

SC - a trainable affective response [45] -, is one of the first skills to be developed in compassion and kindness trainings from several Eastern and Western traditions [45]. A future longitudinal study should evaluate the effects of such training in women leaders.

## Conclusion

In conclusion, SC may be an important characteristic for women leaders because of its association with higher sensitivity to emotional stimuli and mindfulness. These skills may allow them to be more aware of others while being less susceptible for stress and depression symptoms.

## Additional file

Additional file 1: The figures of this supplementary material depict the group average (*n* = 46) activation maps for the main contrasts of interest in the current study. All the statistical images were thresholded by using Gaussian random field-based cluster inference with a threshold of Z > 3.09 at the voxel level and a corrected cluster significance threshold of *P* < 0.05. **Figure S1.** Group average activation map for the contrast pleasant>neutral. **Figure S2.** Group average activation map for the contrast unpleasant>neutral. **Figure S3.** Group average activation map for the contrast pleasant>unpleasant. **Figure S4.** Group average activation map for the contrast unpleasant>pleasant. (DOCX 560 kb)

## Abbreviations

BDI: Beck Depression Inventory
BET: Brain Extraction Tool
DLPFC: Dorsolateral prefrontal cortex
DMN: Default mode network
EPI: Echo planar images
ERP: Event-related potential
FEAT: fMRI Expert Analysis Tool
FFMQ: Five Facets of Mindfulness Questionnaire
FLIRT: fMRIB’s Linear Image Registration Tool
fMRI: Functional Magnetic Resonance Imaging
GLM: General linear model
IAPS: International Affective Picture System
MAAS: Mindful Attention Awareness Scale
MVPA: Multivariate pattern analyses
PSS: Perceived Stress Scale
SC: Self-compassion
SCS: Self-Compassion Scale
TSST: Trier Social Stress Test
