# Supplementary material for: Self-compassion is associated with less stress and depression and greater attention and brain response to affective stimuli in women managers
Source: BMC Womens Health. 2018 Nov 27;18:195. doi: 10.1186/s12905-018-0685-y (PMC6258154; doi:10.1186/s12905-018-0685-y)
Supplement: Supplementary file 1 — The figures of this supplementary material depict the group average (n = 46) activation maps for the main contrasts of interest in the current study. All the statistical images were thresholded by using Gaussian random field-based cluster inference with a threshold of Z > 3.09 at the voxel level and a corrected cluster significance threshold of P < 0.05. Figure S1. Group average activation map for the contrast pleasant>neutral. Figure S2. Group average activation map for the contrast unpleasant>neutral. Figure S3. Group average activation map for the contrast pleasant>unpleasant. Figure S4. Group average activation map for the contrast unpleasant>pleasant. (DOCX 560 kb) [file 12905_2018_685_MOESM1_ESM.docx]

Supplementary Material

The figures bellow depict the group average (n=46) activation maps for the main contrasts of interest in the current study. All the statistical images were thresholded by using Gaussian random field-based cluster inference with a threshold of Z > 3.09 at the voxel level and a corrected cluster significance threshold of P < 0.05.


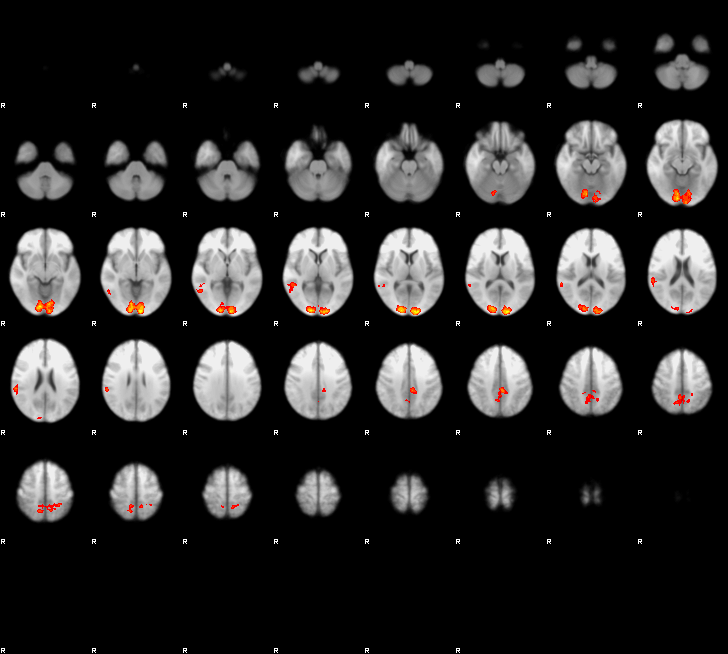


Supplementary figure S1. Group average activation map for the contrast pleasant>neutral.


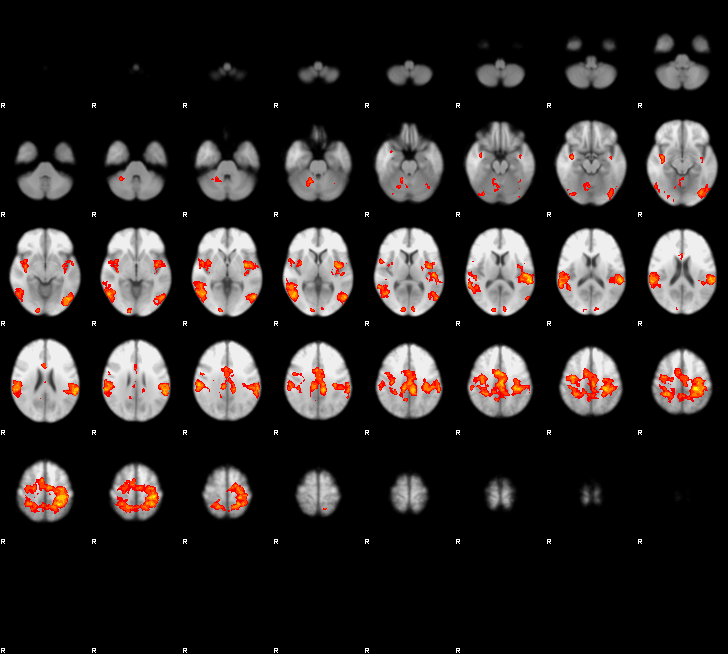


Supplementary figure S2. Group average activation map for the contrast unpleasant>neutral.


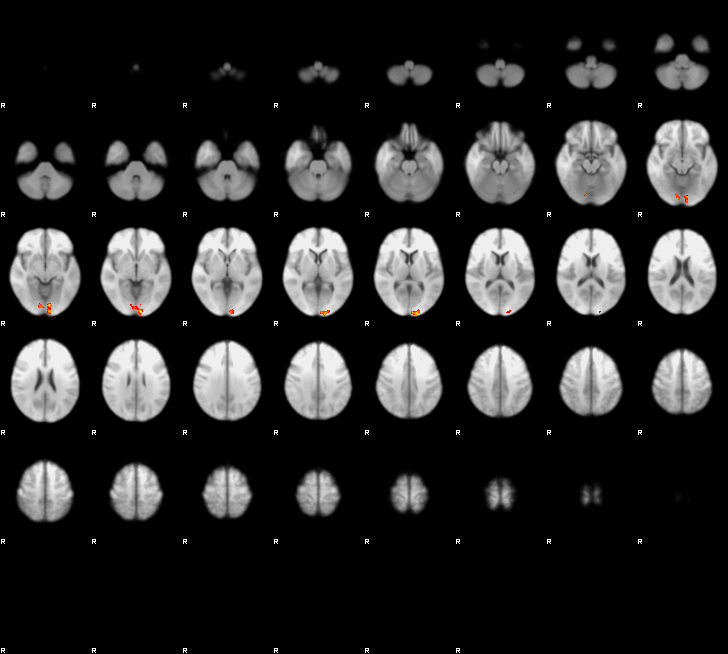


Supplementary figure S3. Group average activation map for the contrast pleasant>unpleasant.


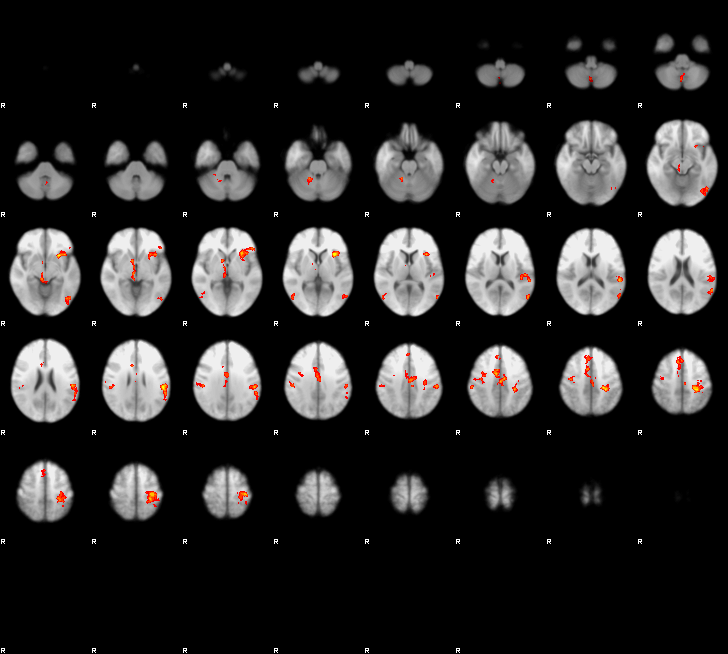


Supplementary figure S4. Group average activation map for the contrast unpleasant>pleasant.
